# Baltimore Medical Association

**Published:** 1878-05

**Authors:** 


### BALTIMORE MEDICAL ASSOCIATION.

Society met on the evening of March 11th, with a large number of members present. After the usual routine of business the appointed subject for the evening was called.

Dr. P. C. Williams was to have read a paper on "Scarlet Fever," but stated that sufficient time had not been given to do justice to the subject; under the circumstances the doctor said that he would claim the attention of the Association for only a brief period, during which he would relate an interesting case of scarlet fever.

The patient was a young girl, aged 4, of previous good health. On Monday morning rose at usual hour; the rainy and unpleasant condition of the weather caused the mother to send for a carriage to take the girl to school; before the carriage arrived she was taken with violent headache and vomiting; the mother sent for Dr. Williams, who responded to the call at once, found the patient in the condition described previously, immediately suspected scarlet fever, prescribed for the patient, and left word that he would call in a few hours.

Tuesday, saw the case again, proceeding as well as possible, the vomiting persisting, and toward night the eruption vanished. Used wet blankets, hot whisky punches, prussic acid, and cupping on the spine.

Wednesday, patient uncomfortable; at night, between ten and eleven o'clock, a stripe of a purple color made its appearance. Should have stated that he had called Prof. Christopher Johnston in consultation in the morning, and when this stripe made its appearance, sent for him again. They noticed a white streak on the face also; the heart beat very feeble; gave stimulants without effect; resorted to the hypodermic use of whisky; the patient got quite comfortable. In the meantime, a most offensive discharge made its appearance from the nostrils, fauces, etc., which increased towards morning.

Thursday morning, met Prof. Johnston, who gave up the case as hopeless, and ceased his visits. Determined to do whatever was possible to relieve the little sufferer, prescribed salicylic acid, 10 grains, every three hours, mixed in a solution of liquor ammoniæ acetatis. In three hours, saw the patient, and found the fetor much less. In six hours, discharge ceased. Remained all night with the case.

Friday, sent for Prof. Johnston, who was much gratified at the change in the patient, who has progressed favorably since that time.

Dr. Williams, on remarking on the case, thought that the patient went through three critical periods:

1. In disappearance of the eruption relieved by baths, cups, etc.

2. Debility and excessive prostration, combatted by hypodermic injection of whisky.

3. The suppuration and fetid discharge from nostrils and fauces, relieved by the use of salicylic acid, which Dr. Williams has used for the first time in this class of cases, and on that account could not claim it as a specific in this disease.

Dr. Gilman expressed himself gratified at the relation of the case by Dr. Williams, and he heartily endorsed the use of salicylic acid in scarlet fever and diptheria.

Dr. J. R. Uhler said that he thought he was the first physician who had used the hypodermic injection of whisky in Baltimore. It occurred at the Alms House in 1866. He had used it with wonderful results. He also thought that some of the beneficial results following Dr. Williams' treatment resulted from the liquor ammoniæ acetatis.

Dr. Jos. T. Smith asked Dr. Williams how the salicylic acid acted.

Dr. Williams thought that it acted directly on the fauces and inflamed surfaces, and also on the blood, as it caused a very rapid disappearance of the peculiar mahogany color of the skin. Dr. Williams also expressed himself in favor of frequent and long visits to ill patients, and in many cases remaining all night, giving the medicine personally, and said he certainly could refer to cases where his personal attention had saved the life of the patient.

On motion, the subject was dropped, and the executive committtee reported that Dr. Quinan would lead at the next meeting, and Dr. I. E. Gibbons at the first meeting in April.

On motion, society adjourned.

